# Patterned immobilization of polyoxometalate-loaded mesoporous silica particles via amine-ene Michael additions on alkene functionalized surfaces

**DOI:** 10.1038/s41598-023-50846-2

**Published:** 2024-01-13

**Authors:** Bingquan Yang, Pierre Picchetti, Yangxin Wang, Wenjing Wang, Christoph Seeger, Kliment Bozov, Sharali Malik, Dennis Mallach, Andreas H. Schäfer, Masooma Ibrahim, Michael Hirtz, Annie K. Powell

**Affiliations:** 1 Institute of Nanotechnology (INT), Karlsruhe Institute of Technology (KIT), 76344 Eggenstein-Leopoldshafen, Germany; 2 Karlsruhe Nano Micro Facility (KNMFi), Karlsruhe Institute of Technology (KIT), 76344 Eggenstein-Leopoldshafen, Germany; 3 Institute of Biological and Chemical Systems – Functional Molecular Systems (IBCS-FMS), Karlsruhe Institute of Technology (KIT), 76344 Eggenstein-Leopoldshafen, Germany; 4 College of Materials Science and Engineering, Nanjing Tech University, Puzhu Road(S) 30, 211816 Nanjing, People’s Republic of China; 5 Institute of Inorganic Chemistry (AOC), Karlsruhe Institute of Technology (KIT), Engesserstraße 15, 76131 Karlsruhe, Germany; 6 Institute for Quantum Materials and Technologies (IQMT), Karlsruhe Institute of Technology (KIT), 76344 Eggenstein-Leopoldshafen, Germany; 7 nanoAnalytics GmbH, Heisenbergstraße 11, 48149 Münster, Germany

**Keywords:** Surface patterning, Surface assembly, Organometallic chemistry, Nanoparticles

## Abstract

Polyoxometalates (POM) are anionic oxoclusters of early transition metals that are of great interest for a variety of applications, including the development of sensors and catalysts. A crucial step in the use of POM in functional materials is the production of composites that can be further processed into complex materials, e.g. by printing on different substrates. In this work, we present an immobilization approach for POMs that involves two key processes: first, the stable encapsulation of POMs in the pores of mesoporous silica nanoparticles (MSPs) and, second, the formation of microstructured arrays with these POM-loaded nanoparticles. Specifically, we have developed a strategy that leads to water-stable, POM-loaded mesoporous silica that can be covalently linked to alkene-bearing surfaces by amine-Michael addition and patterned into microarrays by scanning probe lithography (SPL). The immobilization strategy presented facilitates the printing of hybrid POM-loaded nanomaterials onto different surfaces and provides a versatile method for the fabrication of POM-based composites. Importantly, POM-loaded MSPs are useful in applications such as microfluidic systems and sensors that require frequent washing. Overall, this method is a promising way to produce surface-printed POM arrays that can be used for a wide range of applications.

POMs are anionic oxo-clusters of early transition metals in their high oxidation states, which display unique chemical and physical properties on the basis of their structures, composition, sizes, rich redox chemistry and charges^1,2^. Prominent POM structures are the Keggin-type structure, [XM_12_O_40_]^n−^, and the Wells–Dawson-type structure, [X_2_M_18_O_62_]^n−^^3^. Their properties have been widely exploited in a variety of areas, including catalysis, materials science, photochemistry, molecular magnetism and medicine^4–6^. The high negative charge and multiple oxo-donor sites make them useful multidentate building blocks for constructing transition and lanthanide metal-based cluster complexes. Moreover, their capacity to engage with organic moieties enables the formation of hybrid assemblies with distinctive functionalities^7^. Such assemblies have exhibited significant promise as artificial photosynthesis systems, e.g. in water oxidation^8^ and carbon dioxide reduction^9^. Among the larger investigated POM clusters, the well-known Preyssler*-*type anion^10,11^, of general formula [M^*n*+^P_5_W_30_O_110_]^(15−*n*)−^(abbreviated as {MP_5_W_30_}) is a doughnut-shaped molecule with an inner cavity and 30 terminal O atoms. This polyanion can capture different cations with suitable size such as alkali metals and lanthanide ions (Ln) on the inner surface of the POM. The members of the {MP_5_W_30_} POM family are among the most robust, stable, and processable POM complexes, which are also stable over a wide pH range (pH 1–12)^12^. In addition, their unique biological compatibility and high surface charges result in a flexible architecture for interaction with target substrates, making these POMs a promising molecular sensor material^12,13^.

In this work, we use Preyssler-type POM K_12_[GdP_5_W_30_O_110_]·15H_2_O (hereafter shortened as {GdP_5_W_30_}, structure shown in Fig. 1), synthesized from a Preyssler anion [NaP_5_W_30_0_110_]^14^ in which the central Na^+^ cation was replaced by the Gd^III^ ion under hydrothermal conditions. Gd(III)-substituted POMs play a key role in the development of advanced nanomaterial-based contrast agents for nuclear magnetic resonance (NMR) imaging^15,16^ and thus have great potential for molecular quantum sensing applications^17,18^. These contrast agents are compounds used to enhance the visibility of various tissues, structures, substances, or pathological conditions. They achieve this by emphasizing disparities or boundaries between different substances or tissue types. In the field of imaging, Gd-containing POMs have been found to exhibit a higher *r*_1_ value compared to commercial contrast agents. This improvement is attributed to the significant molecular weight and the rigid framework structure of POMs, which lead to an extended rotational correlation time and an increased *r*_1_ value^19,20^.

**Figure 1:**
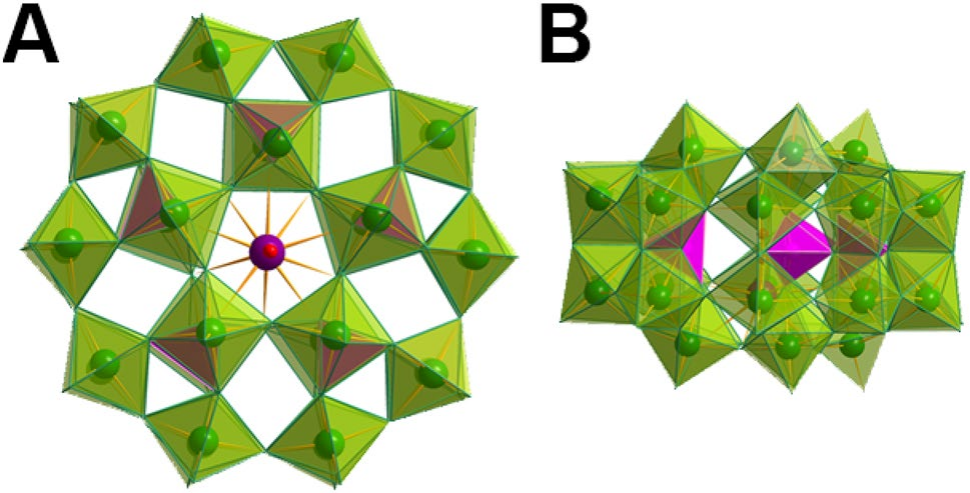
Structure of Preyssler-type POM [GdP_5_W_30_O_110_]^12^ (**A**) Top view, (**B**) side view. Polyhedral color scheme: WO_6_ green, PO_4_ pink. Color scheme for balls: Gd = violet, O = red. The structure was visualized using Diamond 4 software^14^ with a CIF file obtained from single-crystal X-ray diffraction analysis.

However, pure inorganic POMs are usually soluble in many polar solvents, causing difficulties in the recovery, separation, and recycling of the materials^21^. This poses challenges in biomedical and catalysis applications and also for surface-based sensing, where immobilization at high-density would be desirable. To address this problem, various methods for immobilizing POMs into heterogeneous matrices have been developed. The resulting hybrid composites have found applications in various fields, including catalysis^22^, energy conversion and storage^23,24^, molecular sensors and electronics^25^. The immobilization of POMs into cationic silica nanoparticles through covalent or electrostatic binding can yield a novel material, potentially creating a biocompatible nanomaterial with luminescent properties suitable for biosensing and imaging applications^26–28^. In particular, the immobilization of Gd-containing POMs into structured microarrays could enable the use of surface NMR-based methods for molecular quantum sensing, as demonstrated recently in diamond films^29,30^, with the direct use of Gd enhancing the NMR signal instead of nitrogen-vacancy (NV) centers acting as a proxy for readout.

In our attempt to find a straightforward approach to immobilize {GdP_5_W_30_} POM on a glass surface, we decided to use nanometer-sized mesoporous silica particles (MSP)^31–33^ as the POM carriers that, can be covalently tethered onto solid substrates. The choice of MSPs to serve as the POM carrier bears several advantages. First, MSPs can be prepared with tunable porosity (2–50 nm)^34^, sizes and shape^35,36^, and possess high surface areas (up to 1000 m^2^∙g^−1^)^37^. Second, MSPs can be functionalized with a variety of organic functional groups via alkoxysilane chemistry^38,39^. The functionalization of MSP can occur on the outer surface of the particles or the surface of their inner pore walls^40,41^. For example, "clickable" functional groups can be grafted onto silica surfaces by sol–gel chemistry with organoalkoxysilanes bearing amine^42,43^, or azide functional groups^44,45^, which has been used in the past for covalent immobilization of silica and silica-based (nano)particles on solid substrates^46,47^.

## Results and discussion

### Immobilization strategy

**Figure 2:**
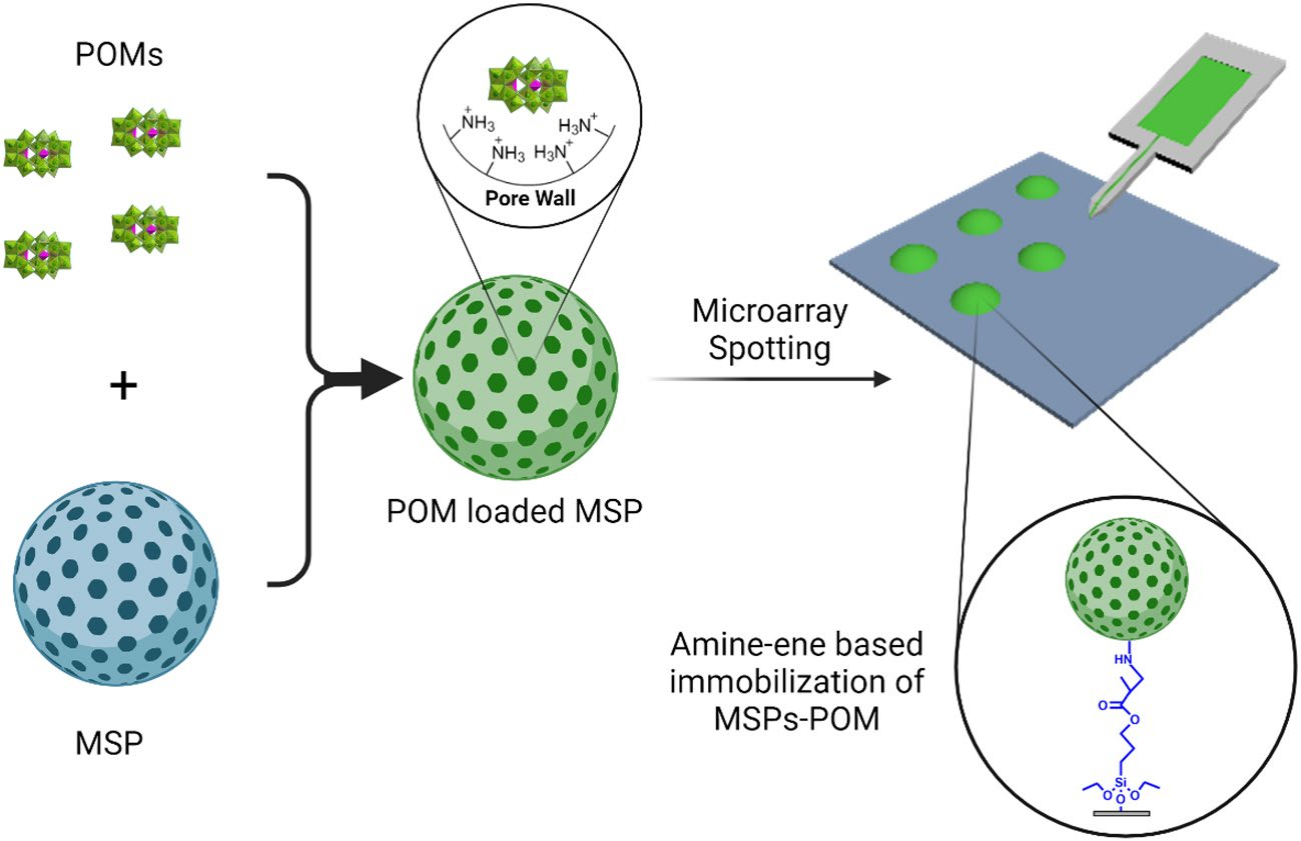
Scheme of the immobilization strategy. MSPs are loaded with POMs to create POM-loaded MSPs. These are then spotted via scanning probe lithography (SPL) methods into microarrays on alkene-bearing surfaces. The POM-loaded MSPs are then immobilized on the surface by amine-Michael addition. Figure created with BioRender.com.

To imprint POMs on surfaces (Fig. 2), we aimed to use MSPs whose pore wall is functionalized with amino groups (MSPs^+^), that are protonated and thus positively charged in an aqueous environment. Due to the size of the mesopores (≈ 3 nm) and the polyanionic nature of the POM, these cargo molecules will be retained in the positively charged pores^48^. Immobilization of the POM-loaded MSP (MSPs-POM) will then be enabled by functionalization of the particle surface with reactive amino groups, which can engage in amine-ene Michael additions to alkene-functionalized surfaces. The advantage of MSPs, therefore, is the possibility to circumvent the use of monofunctionalized POMs^49,50^, whose synthesis requires considerable effort, while providing a generalizable strategy for a wide range of POMs to be immobilized on solid substrates.

### Synthesis of POM-loaded MSPs

To prepare POM-loaded MSPs, first MSPs functionalized with amino groups on the pore wall surfaces (MSPs^+^) were prepared. To this end, pristine spherical nanometer-sized MSPs were synthesized following a previously reported modified Stöber synthesis protocol using the surfactant cetyltrimethylammonium bromide (CTAB) as the structure-directing agent^51^. This synthesis method was chosen because the resulting particles are in the nanometer range, have a spherical shape, have a high specific surface area (1243 m^2^ g^-1^), and are mesoporous (pore diameter ≈ 3 nm)—all properties required for subsequent effective loading of POM. Scanning electron microscopy (SEM) analysis revealed that the pristine MSPs were spherical with an average diameter of (283 ± 25) nm (Fig. S1), which is in good agreement with previously published results.

The pristine MSPs were then passivated on the outer surfaces with a short polyethylene glycol chain (6–9 units) alkoxysilane^52^ and then CTAB was extracted from the mesopores of the particles. Next, the CTAB-free pore walls were covalently grafted with amino groups using (3-aminopropyl)trimethoxysilane (APTMS), resulting in MSPs whose pore walls are functionalized with amino groups (MSPs^+^). The surface passivation and amino functionalization (Fig. S2A) were investigated by zeta potential (Z-pot) analysis (Fig. S2B), which revealed a nearly neutral surface charge (0.4 ± 1.2) mV of MSPs^+^. This reduced negative Z-pot value (for non-functionalized and surfactant-extracted MSP, the Z-pot is around − 30 to − 40 mV) can be explained by the surface passivation that took place, which reduces the amount of deprotonated and negatively charged hydroxyl groups on the surface of the particles, as well as by charge balancing due to the positive R-NH_3_^+^ groups present on the pore entrances.

**Figure 3:**
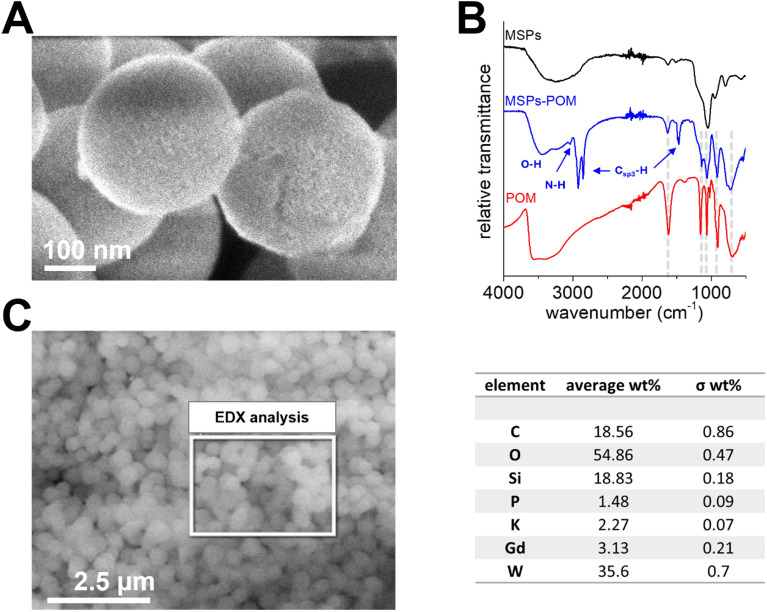
(**A**) SEM image of MSPs-POM. (**B**) ATR-FTIR spectra of MSPs, MSPs-POM, and {GdP_5_W_30_} POM. (**C**) Left: representative SEM image of MSPs-POM and the region (white rectangle) subjected to EDX analysis. Right: atomic weight fractions and errors from EDX analysis of MSPs-POM.

Next, we optimized the procedure for loading MSPs^+^ with POM (MSPs-POM) to eliminate the presence of non-loaded POM crystals. In suboptimal loading procedures, where not all POM was loaded within the pores of MSPs^+^, POM crystals were observed near or on the surface of MSPs, as demonstrated by SEM analysis (Fig. S3A). By optimizing the mass ratio between POM and MSPs^+^ used for the loading and adding several washing steps with EtOH and water, we were able to obtain MSPs-POM (Figs. 3A and S3B), which showed no crystalline POM precipitates (MSPs-POM). Z-pot measurements on MSPs-POM indicated more negative values compared to MSPs^+^ particles, suggesting successful adsorption of POMs within the pores of the particles (Fig. S2B). Attenuated total reflectance Fourier infrared spectroscopy (ATR-FTIR) recorded on MSPs-POM (Fig. 3B) shows the characteristic POM-related transmission bands supporting the presence of POM in the mesopores of the silica nanoparticles (gray background). The FTIR spectrum of MSPs-POM exhibits vibrational bands within the 1200–400 cm^-1^ region, which closely resemble the characteristic bands found in the pristine POM. POMs feature unique metal–oxygen vibrational modes within the fingerprint region. The distinctive peaks at 1134 and 1054 cm^-1^ are attributed to the P–O vibrations of {GdP_5_W_30_} POM. The peak at 907 cm^-1^ could be assigned to terminal **ν**_as_(W══O_t_) vibration. The features around 709 cm^-1^ could be attributed to the edge-sharing **ν**_as_(W–O–W). All these bands are considered as pure vibrations of the POM skeleton. The peak at 1629 cm^-1^ can be attributed to the δ(O–H) of the lattice water molecules. The broad transmission band at 3441 cm^-1^ can be attributed to the presence of -OH groups on the outer surface of MSPs-POM, while the broad bands in the range between 3308 and 3010 cm^-1^ can be attributed to the presence of R-NH_2_/R-NH_3_^+^ groups^53,54^. The three relatively sharp transmission bands at 2917, 2840, and 1460 cm^-1^ are representative of the C_sp3_-H stretching vibration of the organic functional groups of the MSPs-POM particles, *i.e.*, the PEG caps and the condensed APTMS groups^55^. Energy dispersive X-ray (EDX) analysis (Figs. 3C and S4) also confirmed that the POM is colocalized with the particles.

**Figure 4:**
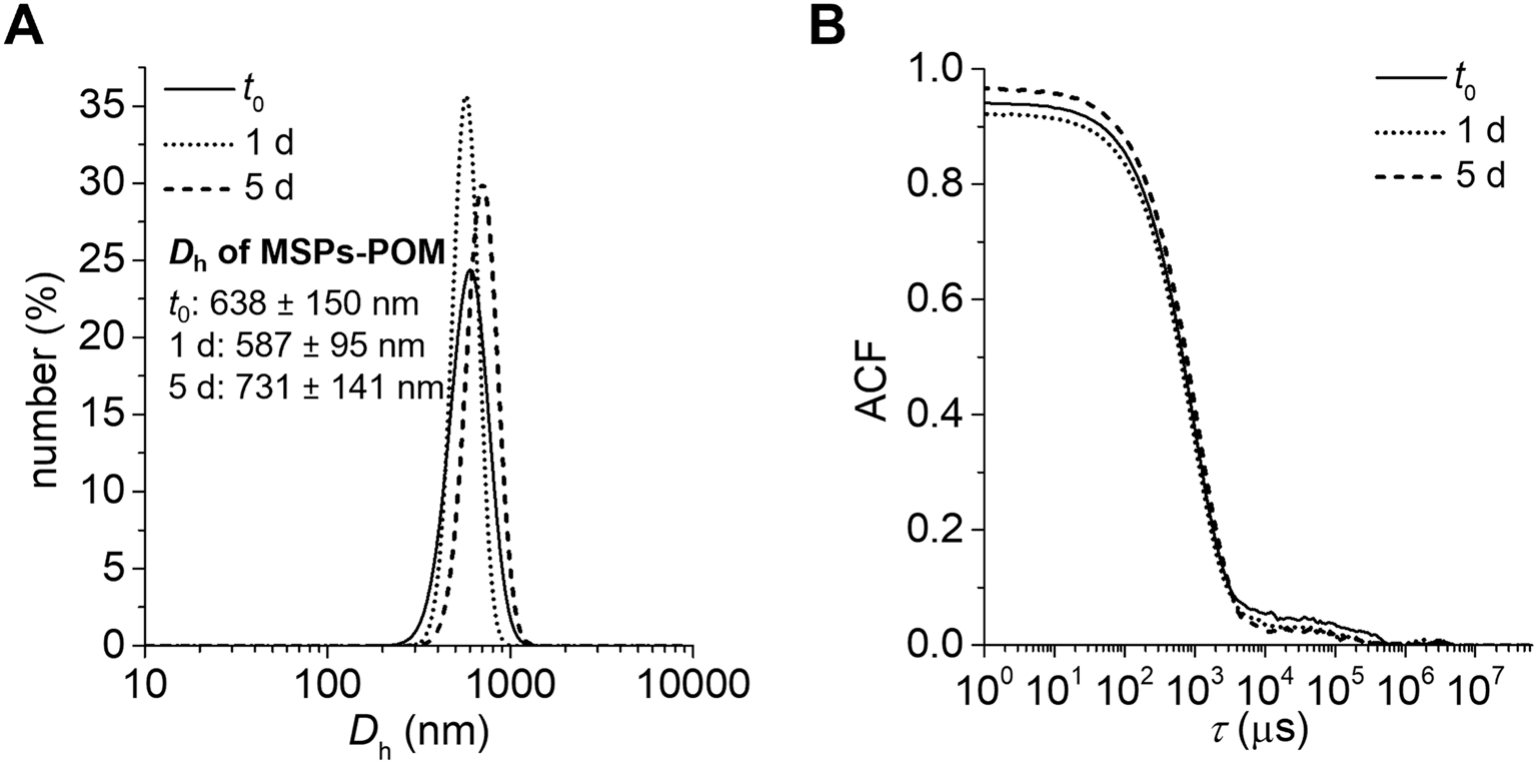
(**A**) Number intensity weighted DLS size distribution of MSPs-POM in PBS buffer (10X, pH 7.5 at 37 °C) and (**B**) corresponding autocorrelation functions (ACF) over 5 days.

To evaluate the stability of MSPs-POM under physiological conditions, their hydrodynamic diameter (*D*_h_) was monitored over time in 10X phosphate salt buffer (PBS, pH 7.4 at 37 °C). Figure 4 shows the DLS size distribution of the particles at time points 0, 1 day, and 5 days. The size distribution remains little affected over 5 d, indicating that both the spherical shape and size of the particles remain intact under physiological conditions. The stability of the particles is achieved by covalent surface passivation of the terminal silanol groups of the silica with PEG silane, which effectively prevents hydrolysis of the silica^56,57^. We also measured the Z-pot to determine whether the POMs could leak out of the mesopores over time, which would be indicated by a clear shift to more positive Z-pot values of the particles. However, over 5 days, the zeta potential of the particles dispersed in PBS showed no significant shift towards more positive Z-pot values (Fig. S5), which proves that the POMs stay adsorbed on the silica surface.

### Preparation and characterization of substrate

**Figure 5:**
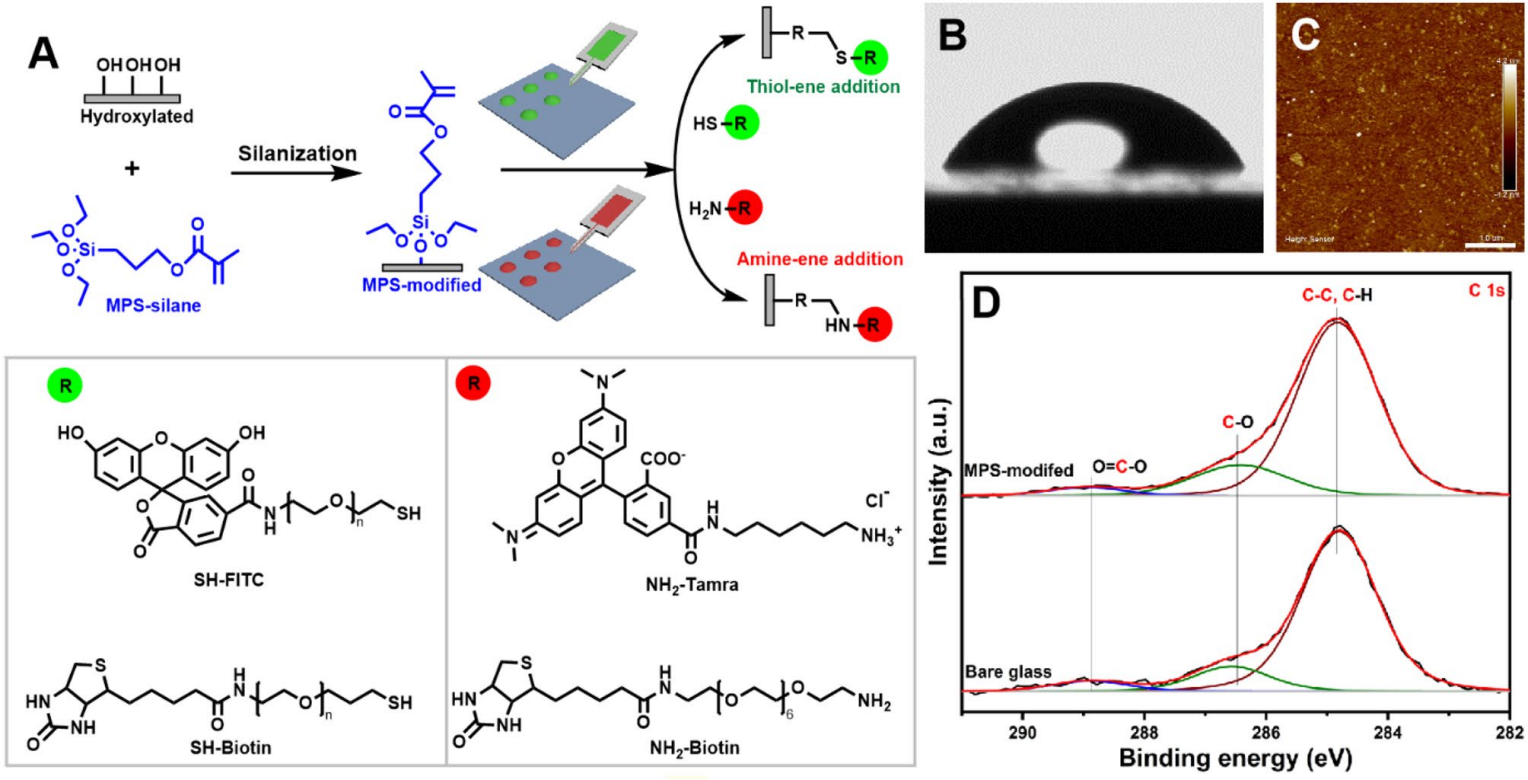
(**A**) Schematic illustration of MPS-modified surfaces functionalization by thiol-ene and amine-ene click reactions and chemical structure of probes used to test the surface reactivity. Results of the characterization of MPS-modified surfaces by (**B**) WCA, yielding (64.3 ± 2.3), (**C**) AFM (Ra = (0.251 ± 0.051) nm), and (**D**) XP spectra at C 1s region of bare glass and MPS-modified surfaces.

The alkene-bearing reactive surfaces were prepared by silanization of 3-methacryloxypropyltrimethoxysilane (MPS-silane) on hydroxylated glass substrates, which can participate thiol-ene and thiol-amine click reactions (Fig. 5A)^58,59^. To achieve efficient silanization, the cleaned glass coverslips were treated with oxygen plasma to remove remaining contaminants and to endow a high density of hydroxyl groups on the glass surfaces. Then, the activated glass slips were immersed in an MPS-silane solution in toluene to obtain the MPS-modified substrates. The characterization of MPS-modified substrates was carried out using atomic force microscopy (AFM), water contact angle (WCA), and x-ray photoelectron spectroscopy (XPS). The results are shown in Fig. 5B–D.

The glass coverslips usually show a WCA at around 43° (Fig. S6A), while just after plasma treatment, the value declines to around 0° (Fig. S6B) due to the densely grafted hydroxyl groups. As shown in Fig. 5B, after silanization of the plasma-treated glass surface with MPS-silane the WCA increases to a higher value at around 64.3°, which is also an indication of a successful silanization process. The mean surface roughness (Ra) of silanized surfaces obtained by AFM is around 0.251 nm, which is consistent with former reports concerning self-assembled monolayers (SAMs)^60,61^. AFM images of a glass coverslip before and after silanization are given in Figs. S6C and 5C, respectively. To further confirm successful silanization, high-resolution XPS (HR-XPS) was implemented on MPS-modified and bare glasses for comparison, the corresponding spectra at the C 1s region are illustrated in Fig. 5D. The deconvoluted HR-XP spectra exhibit three peaks a 284.8 eV, 286.5 eV, and 288.9 eV, which are derived from (C–C, C–H), (C–O), and (O–C=O), respectively. Comparing the XP spectra of the bare glass and MPS-modified samples, the ratio of (C–O):(C–C, C–H) increases from 0.21 to 0.35, which indicates the formation of the MPS-modified SAM.

**Figure 6:**
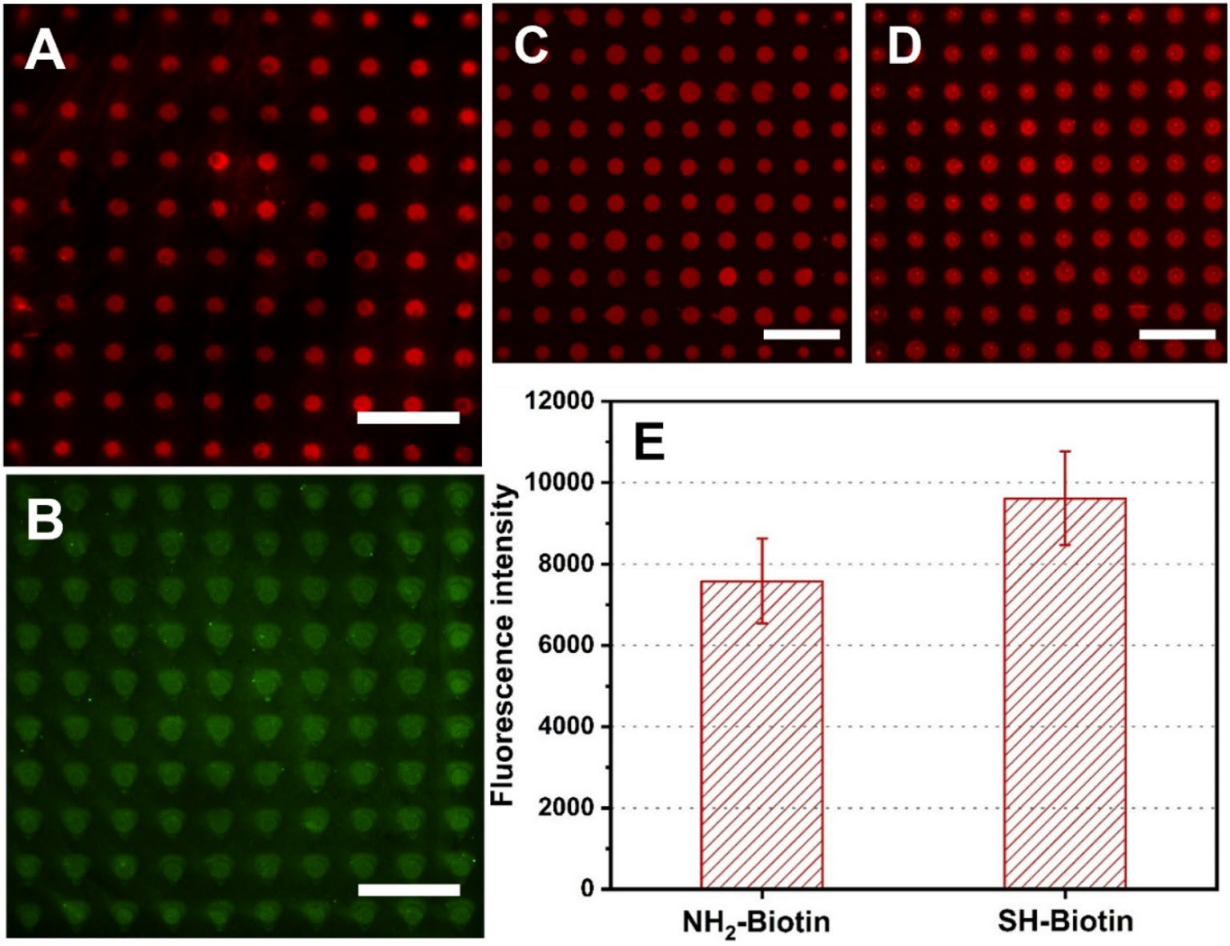
Fluorescent images of microarrays for (**A**) NH_2_-Tamra (exposure time 300 ms) and (**B**) SH-FITC (exposure time 1 s) spots after finished click-reactions and washing of MPS-modified surfaces. Fluorescent images of (**C**) NH_2_-Biotin and (**D**) SH-Biotin spotted MPS-modified surfaces after incubation of SA-Cy3 (Exposure time 500 ms). (**E**) Fluorescence intensity of SA-Cy3 coupled patterns collected from images in C and D. Scale bars equal 100 μm in all images.

To validate the reactivity of the MPS-modified glass substrates and probe the possibility of creating microarrays via microchannel cantilever spotting (µCS) on these, small fluorescent molecules with either a thiol or amine moiety were chosen as test probes, namely NH_2_-Tetramethylrhodamine (NH_2_-Tamra) and SH-Fluorescein (SH-FITC) (Fig. 5A). µCS works by loading a microchannel cantilever with a µL amount of ink containing the desired molecules, and then bringing cantilever repeatedly into contact with the substrate in a scanning probe lithography (SPL) setup allowing for high positioning control, as well as controlled humidity and dwell time. Each spot in the resulting droplet microarrays can be regarded as a microreactor permitting click reactions between the alkene terminal groups and thiol or amine moieties to take place. After the desired incubation time, excess ink can simply be washed away from the surface. As shown in Fig. 6A, the NH_2_-Tamra firmly binds to the MPS-modified surface as the fluorescent pattern is strongly visible in the red channel. Similarly, the SH-FITC also works well with MPS-modified surfaces as shown in Fig. 6B. Therefore, we confirm that the amine and thiol moieties all bind well to the alkene functionalized surface.

As a final test of reactivity and to allow a direct comparison of binding efficiency between thiol-ene and amine-ene routes, two biotinylated compounds, NH_2_-biotin and SH-biotin, were printed. When incubated with Cy3 conjugated streptavidin (SA-Cy3), the printed microarrays can bind the SA-Cy3 via the strong affinity between biotin and avidin^62^, and the binding density of the two microarrays can be directly compared over fluorescence intensity^60,63,64^. After incubation of bovine serum albumin (BSA) for blocking unspecific binding followed by incubation of SA-Cy3 (both in phosphate buffered saline (PBS)) on the biotinylated arrays, the biotin spots are easily readout on a fluorescent microscope as shown in Fig. 6C and D. The corresponding fluorescence intensity comparison is shown in Fig. 6E. The yield of the thiol-ene click route (9620.86 ± 1148.15) a.u. is about 21% higher than amine-ene route (7585.39 ± 1046.44) a.u. from the fluorescence, though both routes appear similar within the fluctuations, both routes deliver a comparable and stable binding to the surface.

### Patterned immobilization of MSPs-POM

**Figure 7:**
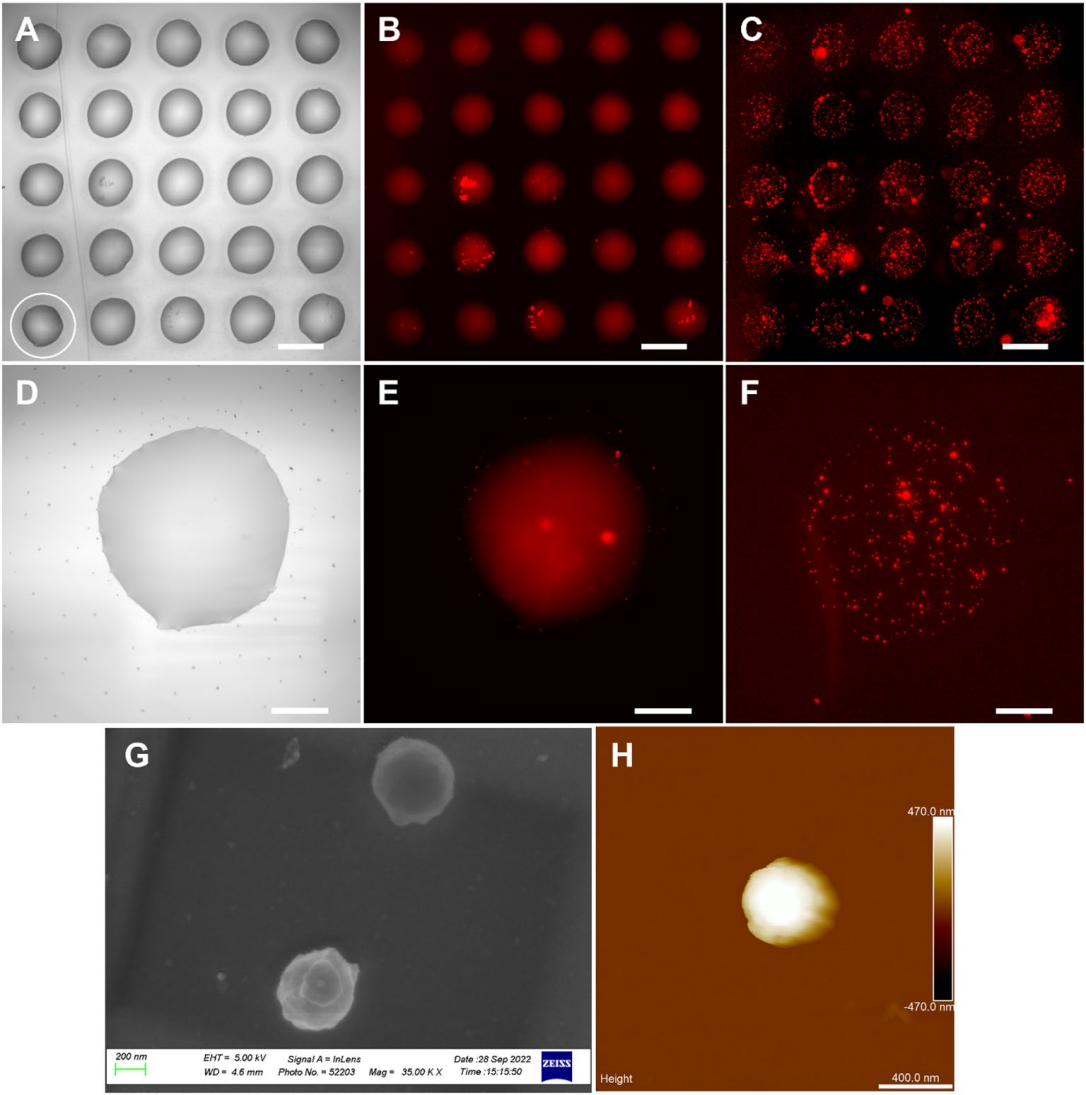
Patterned immobilized MSPs-POM. (**A**) Bright field and (**B**) fluorescence microscopy image of capillary printed microarrays of MSPs-POM ink, before the heating and washing step. (**C**) Fluorescence microscopy image of the microarray after washing. Images of the droplet circled in A, in (**D**) bright field, and fluorescence microscopy images (**E**) before and (**F**) after the heating and washing step. (**G**) SEM image of two immobilized MSPs-POM particles. (**H**) AFM image of an immobilized MSPs-POM particle. Scale bars in (**A–C**) represent 200 µm, in (**D–F**) 50 µm, in (**G**) 200 nm, and in (**H**) 400 nm.

After having obtained amine-functionalized MSPs-POM (H_2_N-MSPs-POM, see Materials and methods) and the matching alkene-functionalized glass surface, capillary spotting was adopted to generate microarrays with MSPs-POM ink. The glass capillary tip has a larger aperture compared to µCS tips and can be tailored to different opening sizes, making it better suited for nanoparticle spotting. After spotting, the obtained microarrays were incubated at 37°C to complete the click-reaction and then washed with water to remove the excess ink. The results of the capillary spotting-based microarray are shown in Fig. 7.

Directly after spotting, the microarray droplets are visible in bright-field and (due to labeling of the MSPs-POM with Cy3) fluorescence microscopy images (Fig. 7A, B). After the click-reaction had taken place and washing, the microarray is not visible anymore in the bright-field, but the fluorescence images revealed the immobilization of MSPs-POM (Fig. 7C). Close-up of individual immobilized MSPs-POM particles were obtained with SEM (Fig. 7G) and AFM (Fig. 7H), further confirming the successful binding to the surface and integrity of the modified MSPs-POM.

## Conclusions

Our proof-of-concept study shows, that the loading of MSPs with POM in conjunction with SPL techniques is a viable route to create surface-immobilized microarrays of MSPs-POM. Encapsulation of POM in MSPs increases stability and enables immobilization of a larger amount of POM, which can outperform direct immobilization methods that typically use molecularly dissolved POM to form surface monolayers. The MSPs-POM is stable under physiological conditions for several days and can therefore be used also for applications in cell cultures. The microarray spotting approach of POMs presented here paves the way for future surface-based applications of POM composites. In particular, the Gd(III)-substituted POM presented here has potential implications for surface microarray-based sensing via surface NMR, acting as a strong molecular NMR chemosensor. The approach is versatile and can, for example, be easily transferred to the loading of other POMs for specific applications. Keeping in mind the capabilities of SPL techniques, in particular the possibility of multiplexing (i.e., deposition of different materials within the same micropatterns) and highly localized deposition (e.g., to deliver functional materials exclusively to prestructures and microdevices like electrodes or sensor devices)^65^, complex functional constructs can be fabricated. Taken together, we think that this MSPs-POM offers an attractive for the immobilization of functional POMs into surface-bound patterns for future applications in biomedical sensing and catalysis.

## Materials and methods

### Chemicals

All solvents for synthesis were purchased from VWR and used as received. Water (18.2 MΩ∙cm) was collected from an Arium (Sartorius) laboratory-grade water purification system. Gadolinium(III) nitrate hexahydrate (Gd(NO_3_)_3_·6H_2_O), sodium tungstate (Na_2_WO_4_) and potassium chloride (KCl) were purchased from VWR. Hexadecyl-trimethylammonium bromide (CTAB, 99 + %) was purchased from ACROS ORGANICS. 2-methoxy(polyethyleneoxy)propyltrimethoxysilane (90%, 6–9 PEG units) was purchased from ABCR Chemie. Triethylamine (TEA, > 99.5%) and hydrochloric acid (HCl, 32 wt. % in H_2_O) were purchased from Merck. Sulfo-cyanine3 (Cy3) NHS ester (95%) was purchased from Lumiprobe. For substrate preparation and printing, Toluene, chloroform, ethanol, dimethyl sulfoxide (DMSO), and glycerol were purchased from Sigma-Aldrich (Germany). Tamra amine, 5-isomer (NH_2_-Tamra) was bought from Lumiprobe GmbH (Germany). Thiol-PEG-Biotin, MW 2000 (SH-Biotin) and Fluorescein-PEG-Thiol (SH-FITC) were obtained from Nanocs Company (USA). Biotin-dPEG_7_-NH_2_ (NH_2_-biotin), streptavidin Cyanine-3 (SA-Cy3), 3-methacryloxypropyltrimethoxysilane (MPS-silane), bovine serum albumin (BSA), and phosphate-buffered saline (PBS) were obtained from Sigma-Aldrich (Germany). A 10X PBS solution contains NaCl: 1.37 M, KCl: 27 mM, Na_2_HPO4: 100 mM, KH_2_PO_4_: 18 mM.

### POM synthesis

All reactants and solvents were of commercially available grade and used without any further purification and all reactions were carried out under aerobic conditions. Synthesis of K_12.5_Na_1.5_[NaP_5_W_30_O_110_]·15H_2_O abbreviated as {NaP_5_W_30_}-POM: This POM ligand was synthesized according to published procedure^12^. Initially, 33 g (100.1 mmol) of sodium tungstate was dissolved in 30 mL of water in a beaker. Subsequently, 26.5 mL of 85% phosphoric acid was added with stirring. Once a clear solution was obtained, it was transferred into autoclaves and prepared for solvothermal synthesis. The autoclaves, containing the reaction solution, were then heated in the oven at 120 °C overnight. After cooling, 15 mL of water were added, and 10 g of KCl were introduced to the solution. A white-yellow precipitate formed, which was filtered off and washed with a 2 M potassium acetate solution and methanol. The resulting white-yellow solid was subjected to two rounds of recrystallization in water to eliminate the {P_2_W_18_}-POM side-product. K_12_[GdP_5_W_30_O_110_]·15H_2_O (abbreviated to {GdP_5_W_30_} in the main text) was prepared following a previously described method with slight modification^12^. Briefly, 1 g (0,12 mmol) of the {NaP_5_W_30_}-POM ligand was dissolved in 12 mL H_2_O and heated to 65 °C while stirring. In the process, the POM dissolved completely. A solution of 3 mL H_2_O and 2 equation  (0,24 mmol) Gd(NO_3_)_3_·6H_2_O was prepared. This solution was added in small portions to the POM solution with stirring. A suspension formed which was placed into the autoclave. This was heated in the oven at 180°C overnight. After the solution had cooled to room temperature, the product was isolated by the addition of 4 g of solid KCl. The precipitate formed was isolated from the mother liquor by filtration and dried in air. On drying, the product was characterized by IR spectroscopy (Fig. S7).

### Preparation of MSPs with amine-groups functionalized pore walls (MSPs^+^)

Pristine MSPs were prepared according to a previously described procedure^51^. Briefly, to a stirring dispersion of CTAB (100 mg, 0.27 mmol) in an H_2_O/EtOH mixture (60/30 v/v, 90 mL), containing NH_3_ (28 wt%, 710 µL), TEOS (250 µL, 1.13 mmol) was added. The resulting reaction mixture was stirred for 16 h at room temperature. Subsequently, 1/3 of the particle dispersion was taken and used for further functionalization steps. To this dispersion of MSPs (30 mL), 2-methoxy(polyethyleneoxy)propyltrimethoxysilane (183.0 µL; 0.38 mmol) was added and the reaction mixture was stirred for 4 h at 60 °C. Subsequently, the particles were recovered by centrifugation (20 min, 3500 rpm) and washed with EtOH (20 mL, 2x) by cycle sonication and subsequent centrifugation.

The surfactant was removed from the pores of the MSPs by extraction in EtOH. For this purpose, the particles were dispersed in EtOH (50 mL), diluted HCl (50 µL; 250 µL HCl diluted in 2 mL EtOH) was added, and the dispersion was refluxed for 12 h. The particles were collected by centrifugation and washed by sonication and centrifugation in EtOH (50 mL, 2×). The collected precipitate was refluxed again in EtOH as described above, but without HCl and for a time of 4 h. Subsequently, the particles were washed with EtOH (50 mL, 2×).

To functionalize the surfactant-free pore walls of the particles, the surfactant-extracted MSP were dispersed in EtOH (10 mL) and (3-aminopropyl)trimethoxysilane (66.0 µL, 0.38 mmol) and triethylamine (66 µL, 0.47 mmol) were added. The resulting reaction mixture was stirred at room temperature for 12 h. The particles were then collected by centrifugation and washed by sonication and centrifugation with EtOH (20 mL, 2×) and finally air dried.

### POM loading of MSPs^+^ (MSPs-POM)

To determine the optimal loading process in which no POM crystals are visible on the surface of the particles or next to the nanoparticles, we developed an optimized loading and washing process. To a dispersion of MSPs^+^ (10 mg) in water (1 mL) POM (40 mg) was added and the resulting mixture was briefly sonicated and stirred at room temperature for 12h. The particles were then collected by centrifugation and subsequently washed with EtOH (1×) and finally with H_2_O (5×).

### MSPs-POM stability tests

In a glass vial (2 mL) with a magnetic stirrer, MSPs-POM was dispersed in 10X PBS buffer (pH = 7.5) by brief sonication (c_particles_ = 0.01 mg·mL^−1^). The resulting particle dispersion was sealed and stirred at 37 °C for 5 days. Over time, aliquots were taken and used to measure the DLS and Z-potential of the sample.

### Dynamic light scattering (DLS)

The measurements were performed using a Malvern ZetaSizer Nano instrument. The intensity of the scattered light was measured at a fixed angle (173°). The wavelength of the laser light used for the light scattering experiments was 633 nm. Data analysis was performed according to standard procedures using the Malvern software (v3.30, https://www.malvernpanalytical.com/). Briefly, the decay rates were determined by the following relationship, where η is the viscosity of the medium, D_h_ is the hydrodynamic diameter, and θ is the scattering angle.

$${\Gamma } = {\text{D}}_{{\text{h}}} \cdot \left( {\frac{{4{{\uppi \upeta }}}}{{\uplambda }}{ } \cdot \sin \left( {\frac{{\uptheta }}{2}} \right)} \right)^{2}$$

The method of cumulants was used to fit the autocorrelation function, which in turn allows the determination of the diffusion coefficient (d), from which the hydrodynamic diameter (D_h_) of the aggregates is calculated using the Stokes–Einstein equation (see below), where k_b_ is the Boltzmann constant, T is the temperature, and η is the viscosity of the medium.

$${\text{D}}_{{\text{h}}} = \frac{{{\text{k}}_{{\text{b}}} \cdot {\text{T}}}}{{3 \cdot {\uppi } \cdot {\upeta } \cdot {\text{d}}}}.$$

### Z-potential measurements

The measurements were performed using the Malvern ZetaSizer Nano instrument. A viscosity of μ = 0.8984 cP and a dielectric constant of ε = 79 and a refractive index of 1.33304 were used. Analysis of data was performed utilizing Zetasizer Software 6.12 (Malvern Instruments GmbH, Germany) based on the model of Smoluchowski.

### Surface amino functionalized and fluorescent MSPs-POM (H_2_N-MSPs-POM)

First, the Sulfo-Cy3 alkoxysilane used for fluorescent labeling of the particles is prepared. For this purpose, APTMS (8 µmol, from a 100 µM stock solution in dry DMSO) is added to a solution of Sulfo-Cy3-NHS ester (0.6 mg, 0.8 µmol) in dry DMSO (50 µL) in a glass vial, mixed briefly, and allowed to react for 30 min at room temperature. A dispersion of the MSPs-POM (10 mg) in toluene (500 µL) was also prepared. To this particle dispersion, the crude SulfoCy3-silane mixture and APTMS (10 µL) were added, and the reaction mixture was stirred for 12 h at 60 °C. The particles were then collected by centrifugation and washed with EtOH until no sulfo-Cy3 was detected in the supernatant of the wash solutions. The particles were then dried under reduced pressure.

### Fourier-transform infrared (FT-IR) spectroscopy

FT-IR spectroscopy was employed to analyze the acquired compounds using the ATR module of a NicoletTM iS50 spectrometer, and the resulting data was visualized using Origin 2018b.

### Preparation of substrates

Glass coverslips (diameter 15 mm, VWR, Germany) were cleaned by sonication in chloroform, ethanol, and water for 5 min each and then dried with nitrogen. Straight after, 5 min of plasma treatment (10 sccm O_2_, 0.2 mbar, and 100 W, ATTO plasma system, Diener electronics, Germany) was done under oxygen. Subsequently, the freshly hydroxylated substrates were immersed in a freshly prepared MPS-silane solution in toluene (1%, v/v) overnight at room temperature, and then the substrates were washed with toluene, ethanol, and water, and then dried under a nitrogen stream. Finally, the MPS-modified glass samples were stored in a desiccator.

### Ink solutions preparation

Ink solutions for click reaction were prepared by mixing thiol-containing or amine-containing compounds in a mixture of DMSO/TEA (10:3, v/v). To avoid fast evaporation of the ink solvent, an amount of 30% (v/v) of glycerol was added to the ink solutions. The final concentration of the ink solutions was 2 mg/mL.

### Microarray patterning

For µCS, the patterns were created on an NLP 2000 instrument (Nanoink, USA), which was equipped with a microchannel cantilever (SPT-S-C30S, Bioforce, Nanosciences, USA). Before loading inks (0.2 μL), the tips were plasma cleaned by oxygen (0.2 mbar, 100 W, 20 sccm O_2_, 2 min) to promote ink transfer, and then the inks were pushed into the microchannel by blowing with a nitrogen stream. All patterning processes (10 × 10 spot array with a pitch of 50 µm) were done at a humidity of 40% and dwell time of 0.5 s. After printing, the samples were heated at 37°C for 30 min before being allowed to rest overnight at room temperature (RT) to complete the click reaction, and then washed with water to remove the excess ink. The biotin-bearing microarrays for the comparison of binding efficiency of the thiol-ene and amine-ene coupling routes were incubated as follows. First, the samples were blocked against unspecific protein binding by incubation with 10% BSA in PBS for 30 min. Then washed by pipetting on and off 30 μL of PBS three times and subsequently incubated with 100 μL of 1 mg mL^–1^ SA-Cy3 in PBS (1:100) at 37 °C for 30 min in a dark environment. Finally, samples were rinsed 3 times with PBS and blown dry under a stream of nitrogen before evaluation in fluorescence microscopy. For capillary spotting, a custom-made setup was used as previously reported for liquid metal deposition^66^, but without connection of a microfluidic pump. In the present study, the capillary tips were simply immersed in a reservoir of the MSPs-POM solution, enabling the loading of ink via capillary forces. Microarrays with MSPs-POM (5 × 5 spot array with 300 µm pitch) were obtained with a glass capillary tip of approx. 100 µm aperture in an NLP 2000 system (Nanoink, USA) under 40% relative humidity with a dwell time for each spot of 1s. The samples were then incubated at 37°C for 3 h and left at RT overnight to complete the click reaction. Finally, excess ink was removed by washing with water.

### Physico-chemical characterization of substrates

The static WCA was measured on an OCA-20 contact angle analyzer (DataPhysics Instruments GmbH, Germany) at room temperature. Concisely, a 3 μL water drop was dispensed on a sample surface, and the measurements were repeated three times for each sample to obtain the means and standard deviations. The roughness and topography of the sample surface was evaluated on an AFM (Dimension Icon, Bruker, Germany), at room temperature in the air in tapping mode (40 N m^-1^, 325 kHz, HQ:NSC15/Al BS, MicroMasch, Germany). Three random positions were scanned for each sample (in 5 × 5 μm), and the original roughness Ra was extracted by the onboard software of the instrument. Apart from this, the analysis of chemical compositions of the surface in each step was identified by X-ray photoelectron spectroscopy (XPS) using a Thermo Scientific K-Alpha system (XPS, Thermo Fisher Scientific, East Grinstead, UK) with a base pressure of about 2 × 10^–9^ mbar. Excitation was done using monochromatic Al-Ka-X-rays. The energy calibration of the system was done according to ISO 15472:2001 using copper, silver and gold reference samples. The transmission function was determined using the build-in thermo standard method on a silver reference sample. Quantification of the measurement results was done using modified scofield sensitivity factors. A 400 µm X-ray spot was used for the analysis. On non-conducting samples, a flood gun was used for compensation of charging. XPS data was processed using the CasaXPS software^67^ (suite version 2.3.25). The energy scale of the spectra was set to 285 eV based on the C–C/C–H part of the C1s signal. A Shirley background was used for the evaluation of the high-resolution spectra.

### Optical imaging

The optical images were captured on a Nikon Eclipse 80i upright fluorescence microscope (Nikon, Germany) equipped with an Intensilight illumination (Nikon, Germany), a Nikon DS Qi2 camera, and FITC and Tamra filters (Nikon Y-2E/C). The microscope collected the fluorescence intensity data with the built-in NIS-element software (Nikon, Germany).

### Electron microscopy

The SEM images were acquired with a Zeiss Ultra-Plus SEM at 3 kV and, 5kV. The EDX measurements were performed with a Zeiss Leo 1530 SEM operating at 20 kV. The EDX data were acquired with Oxford Instruments AZtec software using an Oxford X-Max^N^ 50 detector.

### Statistical analysis

All data shown in this work were described as means ± standard deviations. The values of fluorescence intensity were obtained by an on-board software (NIS Elements AR 5.02.01, Nikon) of the microscope. The original data of WCA and roughness were obtained by measuring 3 random points of each sample, and the means and standard deviations were computed on excel by STDEVA formula.

### Supplementary Information

Supplementary Figures.

## Data Availability

The datasets used and/or analysed during the current study available from the corresponding author on reasonable request.
