# Supplementary material for: Patterned immobilization of polyoxometalate-loaded mesoporous silica particles via amine-ene Michael additions on alkene functionalized surfaces
Source: Sci Rep. 2024 Jan 13;14:1249. doi: 10.1038/s41598-023-50846-2 (PMC10787769; doi:10.1038/s41598-023-50846-2)
Supplement: Supplementary file 1 — Supplementary Figures. [file 41598_2023_50846_MOESM1_ESM.pdf]

Supplementary Information for

Patterned immobilization of polyoxometalate-loaded mesoporous silica particles via amine-ene Michael additions on alkene functionalized surfaces

Bingquan Yang[1,2], Pierre Picchetti[1], Yangxin Wang[3,4], Wenjing Wang[1,2], Christoph Seeger[1], Kliment Bozov[1], Sharali Malik[6], Dennis Mallach[7], Andreas H. Schäfer[7], Masooma Ibrahim[1], Michael Hirtz[1,2]

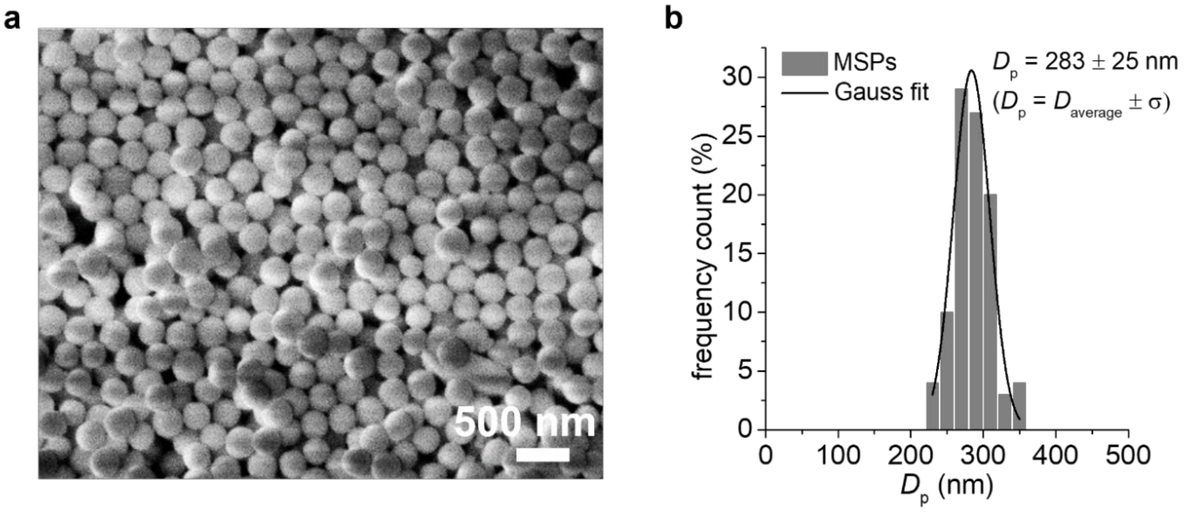

Figure S1. (**A**) SEM image of MSPs. (**B**) Size distribution of MSPs particles calculated from SEM images (N = 100).

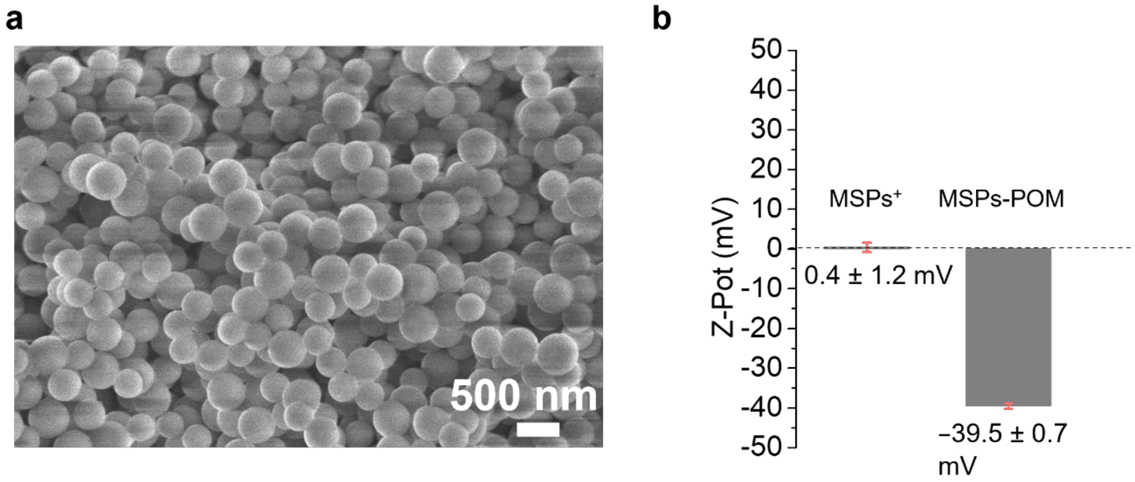

Figure S2. (**A**) SEM image of $MSPs^+$. (**B**) Z-pot analysis of $MSPs^+$ and H_2N-MSPs-POM in water (pH 7.0).

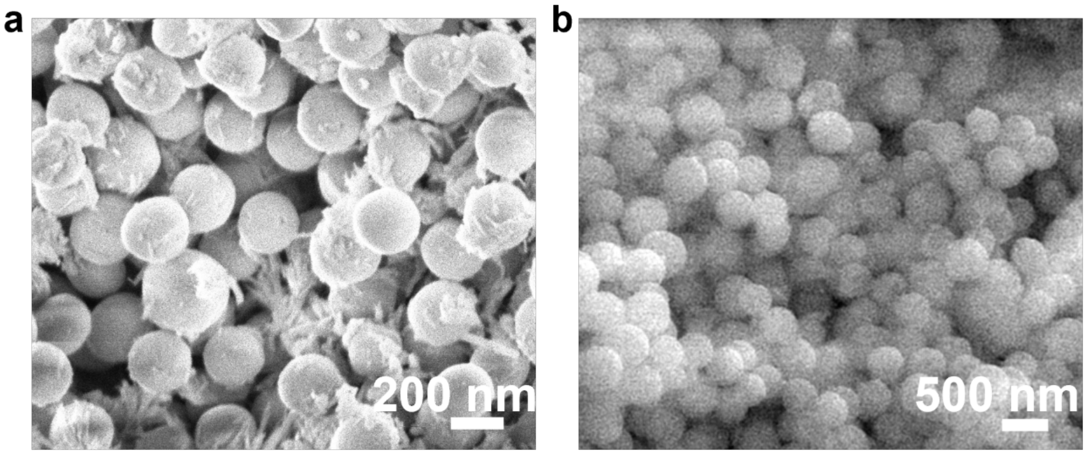

Figure S3. (**A**) SEM image of POM-loaded MSP from a non-optimized loading process. The fibrous structures represent crystalline POM deposits. (**B**) SEM image of MSP-POM particles prepared by the optimized loading method.

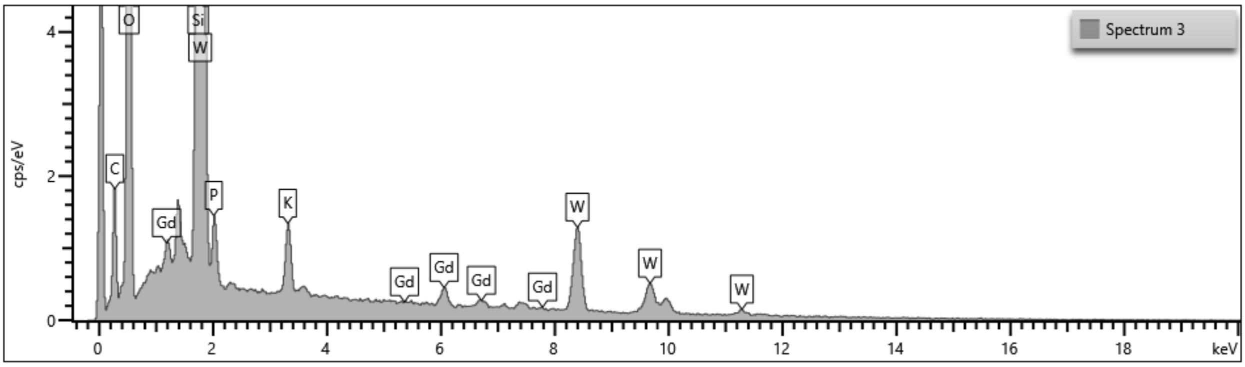

Figure S4. EDX spectrum recorded on MSPs-POM shown in the SEM images of Fig. 3C.

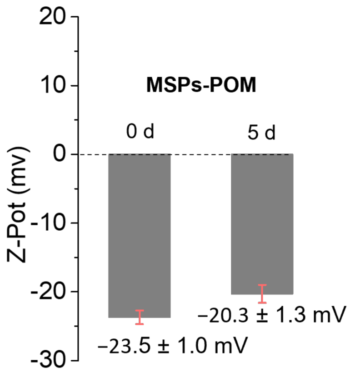

Figure S5. Zeta potential (Z-Pot) of MSPs-POM dispersed in PBS buffer (10X, pH 7.5 at 37°C) before (0 d) and after 5 days (5 d).

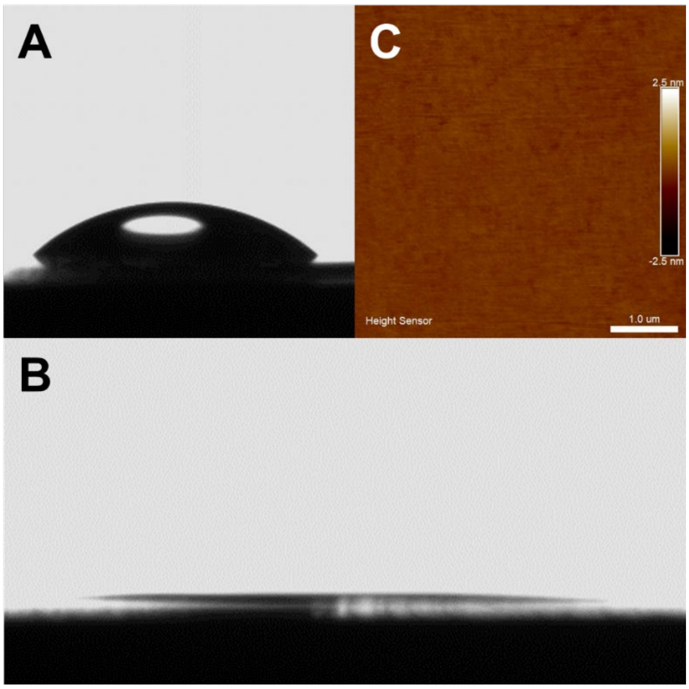

Figure S6. WCA of (**A**) bare glass (43.3 ± 2.6°) and (**B**) plasma treated glass (0°), and (**C**) AFM image of bare glass (0.151 ± 0.037 nm).

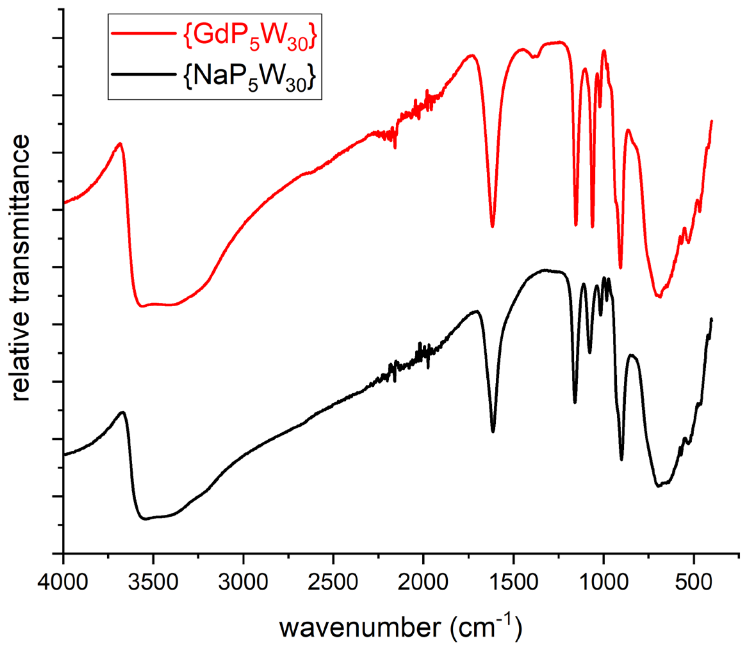

Figure S7. Comparison of ATR-FTIR spectra of $\{GdP_5W_{30}\}$ and $\{NaP_5W_{30}\}$ POM ligand.
